# Differential Gene Expression in Articular Cartilage and Subchondral Bone of Neonatal and Adult Horses

**DOI:** 10.3390/genes10100745

**Published:** 2019-09-25

**Authors:** Ann M. Kemper, Jenny Drnevich, Molly E. McCue, Annette M. McCoy

**Affiliations:** 1 Department of Veterinary Clinical Medicine, University of Illinois College of Veterinary Medicine, Urbana, IL 61802, USA; amkemper@illinois.edu; 2 Roy J. Carver Biotechnology Center, University of Illinois, Urbana, IL 61801, USA; drnevich@illinois.edu; 3 Veterinary Population Medicine Department, University of Minnesota College of Veterinary Medicine, St. Paul, MN 55108, USA; mccu0173@umn.edu

**Keywords:** transcriptome, skeletal development, joints, developmental orthopedic disease, equine

## Abstract

Skeletogenesis is complex and incompletely understood. Derangement of this process likely underlies developmental skeletal pathologies. Examination of tissue-specific gene expression may help elucidate novel skeletal developmental pathways that could contribute to disease risk. Our aim was to identify and functionally annotate differentially expressed genes in equine neonatal and adult articular cartilage (AC) and subchondral bone (SCB). RNA was sequenced from healthy AC and SCB from the fetlock, hock, and stifle joints of 6 foals (≤4 weeks of age) and six adults (8–12 years of age). There was distinct clustering by age and tissue type. After differential expression analysis, functional annotation and pathway analysis were performed using PANTHER and Reactome. Approximately 1115 and 3574 genes were differentially expressed between age groups in AC and SCB, respectively, falling within dozens of overrepresented gene ontology terms and enriched pathways reflecting a state of growth, high metabolic activity, and tissue turnover in the foals. Enriched pathways were dominated by those related to extracellular matrix organization and turnover, and cell cycle and signal transduction. Additionally, we identified enriched pathways related to neural development and neurotransmission in AC and innate immunity in SCB. These represent novel potential mechanisms for disease that can be explored in future work.

## 1. Introduction

Skeletogenesis is a complex process that is tightly regulated via the interactions of various transcription factors, growth factors and cytokines on progenitor cells of mesenchymal (osteoblast, chondrocyte) and hematopoietic (osteoclast) origin [1,2,3]. Long bones are formed via endochondral ossification, the process whereby the cartilage anlage ossifies and becomes bone. Although most skeletal development occurs in the fetus during gestation, endochondral ossification continues in the post-natal period at the growth plate and articular-ephiphyseal cartilage complex until the time of skeletal maturity [3]. In the horse, most growth plates in the distal limb close within the first year of life [4], although skeletal maturity is not reached until six years of age [5].

Our understanding of the genes and pathways involved in normal skeletogenesis has been largely driven by studies of morphogenesis in model species, including mice [6,7], zebrafish [8], chickens [9], and amphibians [10,11], and by investigation of severe skeletal dysplasias in humans [1]. These have identified a number of significant signaling factors involved in cellular differentiation, proliferation, and maturation, as well as the production of extracellular matrix (ECM) proteins. However, our understanding of the process remains incomplete, and there is evidence that there are differences between species [12]. In particular, most investigations in model species are focused on embryonic stages of development, with relatively little work examining endochondral ossification in the post-natal period except in relation to tissue regeneration [7,13] and bone healing (particularly in sheep models) [14,15].

One reason to investigate pathways of skeletogensis in the post-natal period is that this information may be useful in determining pathophysiology and genetic risk factors for various developmental orthopedic diseases. Developmental orthopedic disease (DOD) is an umbrella phrase used to encompass a variety of conditions related to abnormal growth and development of skeletal structures in young animals. Specific conditions that fall within the constellation of DOD include osteochondrosis, subchondral bone cysts, vertebral malformations, angular limb deformities, non-septic physitis, flexural limb deformities, and incomplete ossification of cuboidal bones [16]. Although DOD affects individuals across species, it is particularly prevalent in the horse, where radiographic surveys suggest that 40-60% or more of foals are affected by one or more manifestations of DOD [17]. Risk factors for DOD are thought to be both genetic and environmental (e.g., diet, biomechanical forces/exercise, conformation, in utero exposures, etc.). In the horse, manipulation of diet and exercise have had limited effect in reducing disease prevalence, thus highlighting the importance of genetics in disease development [17,18]. However, despite strong evidence demonstrating heritability (h^2^ = 0.27–0.52 [19,20,21]), specific genes and variants underlying disease risk in the horse are completely unknown.

While derangements of normal developmental pathways are known to underlie various human skeletal disorders that manifest as significant global skeletal abnormalities in affected individuals (e.g., sclerosteosis and achondroplasia [22]), DOD tends to occur only at specific predilection sites, and thus it is unlikely that the same mutations identified in human skeletal dysplasias also underlie these more focal diseases. A more complete understanding of normal skeletal development pathways in the post-natal period will both increase our understanding of this complex process and help to identify biologically compelling candidate genes underlying disease risk. There are almost certainly many pathways involved in the regulation of skeletal development and growth that have yet to be described [23], and we postulate that examination of tissue-specific gene expression in articular cartilage (AC) and subchondral bone (SBC) will provide a tool to help elucidate these novel pathways. The aim of this work, therefore, was to assemble transcriptomes for equine neonatal and adult AC and SCB and to identify genes that were uniquely or differentially expressed in the neonatal tissue compared to the adults, as well as enriched pathways defined by these genes. 

## 2. Materials and Methods 

### 2.1. Tissue Collection and RNA Extraction

Experimental protocols were approved by the appropriate Institutional Animal Care and Use Committees at the University of Minnesota (protocol #1109B04448) and the University of Illinois (protocols #15188 and 18213). AC and SCB were collected from grossly normal metatarsophalangeal (fetlock), tarsocrural (hock), and femoropatellar (stifle) joints of horses euthanized for reasons unrelated to the study and unrelated to pathology of the hind limbs. Euthanasia was performed in all cases following standard clinical protocols of intravenous overdose of pentobarbital (390mg/mL; 1ml/10lbs {1ml/4.5kg}). Adult horses (*n* = 6; 4 males, 2 females) ranged in age from 8–12 years. Foals (*n* = 6; 5 males, 1 female) ranged in age from 1 day to 4 weeks. Several breeds were represented, although the majority were Quarter Horses (Quarter Horse, *n* = 7; Paint, *n* = 3; Morgan, *n* = 1; Friesian, *n* = 1). Samples were collected immediately following euthanasia and were flash frozen in liquid nitrogen or placed in RNAlater (Qiagen, Valencia, CA, USA) at 4 °C for 24–48 h prior to flash freezing; all samples were subsequently stored at −80 °C until processing. Frozen samples were crushed to powder with a mortar and pestle and placed in TRIzol reagent (Invitrogen, Carlsbad, CA, USA) prior to mechanical homogenization for cell lysis. RNA extraction was performed on spin columns using the RNeasy Micro Kit (Qiagen, Valencia, CA, USA) per manufacturer instructions.

### 2.2. RNA Sequencing

In total, 74 tissue samples were collected from the 12 horses and some were merged to create 56 samples for sequencing (Table S1). In four of the adult horses, samples from the three joints (metatarsophalangeal, tarsocrural, and femoropatellar) were pooled prior to sequencing. In all other cases, samples from each joint were sequenced separately; two foals had two AC samples taken from slightly different depths in the femoropatellar joint (superficial {at the joint surface} and deep {adjacent to the SCB} AC) and these were sequenced separately. For SCB, this resulted in a total of 18 sequenced foal samples (from 6 foals) and 10 sequenced adult samples (from 6 adults). Two of the adult AC samples failed (one each from the metatarsophalangeal and femoropatellar joints), resulting in a total of 20 sequenced foal samples (from 6 foals) and 8 sequenced adult samples (from 6 adults) for this tissue. All samples were subjected to standard library preparation and sequencing (100 base-pair, paired-end) on an Illumina Hi-Seq sequencer. The four adult horses (8 samples) in which tissue from the three joints was pooled were sequenced at the University of Minnesota Genomics Center (UMGC) on an Illumina Hi-Seq 2500. The remaining samples were sequenced at the University of Illinois Roy J Carver Biotechnology Center (RJCBC); the first four foals (24 samples) were sequenced on an Illumina Hi-Seq 2500 while the remaining samples (2 adults, 2 foals; 24 samples) were sequenced on an Illumina Hi-Seq 4000. Potential batch effects were accounted for in the statistical methods (below). RNA sequences have been deposited into the National Center for Biotechnology Information (NCBI) Gene Expression Omnibus (GSE135322).

### 2.3. Data Analysis

#### 2.3.1. Alignment and Gene-Level Quantification

Full details of the data analysis methods can be found in Appendix A. In brief, the samples were quality checked using fastp [24] (version 0.19.5), then trimmed using Trimmomatic [25] (version 0.38). Salmon [26] (version 0.11.3) was used to quasi-map reads to the transcriptome from NCBI’s EquiCab3.0 and Annotation Release 103 and quantify the abundance of each transcript, which were summed to gene-level using tximport [27]. At this point, gene counts for the two tissues were analyzed separately. Additionally, gene-level counts were added together for the two foals that had two samples of AC collected from the femoropatellar joint.

#### 2.3.2. Filtering, Clustering and Surrogate Variable Analysis 

While the NCBI EquCab3.0 Annotation Release 103 transcriptome has a total of 29,196 genes, a large proportion of these are not expected to have detectable expression. Genes without at least 1 cpm (counts per million) in at least 2 samples were filtered out, leaving 16,440 genes to be analyzed for differential expression in AC (accounting for 99.92% of reads) and 18,009 genes to be analyzed for differential expression in SCB (accounting for 99.91% of reads). After filtering, TMM normalized log2-based count per million values (logCPM) were calculated using edgeR’s ‘cpm’ function with prior.count=3 to help reduce variability in fold-changes of extremely low expression genes [28]. 

The main variable of interest was age (foals vs adults), with location (metatarsophalangeal vs tarsocrural versus femoropatellar joints) of secondary interest. Since the two adult horses with separate samples for each joint showed very little difference between joints in either tissue (see Appendix A.4), the four adult horses who had their three joint locations pooled per tissue were each assigned to one of the locations to balance out sex and replicate number for the statistical analysis. While this invalidates any within-location adult comparisons, it allows for overall foal vs. adult comparisons, which was the primary variable of interest. Additionally, there were many nuisance variables including individual horse effects, foal age, sequencing year, and sex. Some of the nuisance variables were partially or completely confounded, making it difficult to adjust for them in a traditional manner. Instead, we employed surrogate variables analysis [29,30] that estimated five continuous quantitative variables within each tissue that were added as covariates to the model of age * location to correct for extraneous variation in the samples when testing for differential expression. The effects of surrogate variables can also be removed directly from the normalized logCPM values for visualization purposes, such as multidimensional scaling (MDS) clustering and heatmaps of expression patterns (see Appendix A.5).

#### 2.3.3. Differential Expression Testing

Differential gene expression analysis was performed using the limma-trend method [28,31] on the logCPM values using a model that treated the six different age x location groups as a single factor, plus the 5 surrogate variables. The single factor model gives equivalent results as a traditional 2-factor model, but is easier to make explicit contrasts [32]. Pairwise comparisons were made for within-location age differences (foal vs adult for each joint) and within-age location differences (metatarsophalangeal versus tarsocrural, tarsocrural versus femoropatellar, and metatarsophalangeal versus femoropatellar for each age). Multiple testing correction using the false discovery rate (FDR) method [33] was initially done separately for all nine comparisons. However, due to the large number of expression changes across ages, but the few expression changes across locations, the same raw *p*-value ended up with very different FDR *p*-values in different contrasts. To combat this, the pairwise comparisons were also FDR corrected together using the “global” method so that the same raw *p*-value in all contrasts ended up with the same FDR *p*-value. At a global FDR *p*-value < 0.05, thousands of genes were significantly DE (~2000 in AC, ~7000 in SCB), therefore we used an additional “TREAT” test [34] to select genes that had a |fold-change| (FC) significantly larger than 1.5 prior to functional annotation, which prevents biases towards low expression genes that occur during the common practice of requiring a *p*-value threshold from a regular statistical test of no change in expression and simultaneously a minimum fold-change threshold [28].

#### 2.3.4. Functional Annotation and Pathway Analysis

Gene symbol, gene name, Entrez gene identification, gene ontology (GO) identification, and GO terms for each gene were obtained using Bioconductor’s [35] AnnotationHub [36] web resource to pull the “OrgDb” for *Equus caballus* from NCBI. KEGG pathways for each gene were retrieved directly from http://www.genome.jp/kegg using the KEGGREST package [37].

Entrez gene IDs were converted to protein IDs using NCBIs “Batch Entrez” retrieve function (hittps://www.ncbi.nlm.nih.gov/sites/batchentrez). The associated FASTQ protein sequences were input into the EggNOG database [38] (version 4.5.1) to identify orthologues across species. This allowed consensus gene names and UniProt gene identifiers to be assigned to DE genes rather than the species-specific identifier from the EquCab3.0 annotation. UniProt gene IDs for all DE genes were input into PANTHER [39] (version 14.1). The PANTHER Overrepresentation Test was performed to identify enriched GO-slim terms for molecular function, biological process, and cellular component within the gene set [40]. For all tests, a Fisher’s exact test was used with FDR correction. Significance was set at FDR *p* < 0.05.

Pathway analysis was performed using both PANTHER (Reactome version 65) and the Reactome Pathway Knowledgebase [41]. While PANTHER leverages the Reactome database for its analysis, the two programs employ different algorithms for determining significant overrepresentation (enrichment). PANTHER employs a Fisher’s exact test with FDR correction, while Reactome utilizes a hypergeometric test that produces a probability score that is subsequently corrected for FDR. For both analyses, significance was considered at FDR < 0.05. 

## 3. Results

### 3.1. MDS Clustering

**Figure 1:**
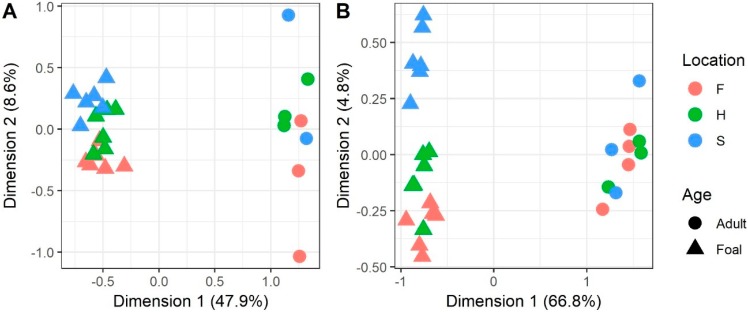
Multidimensional scaling (MDS) was run on normalized gene expression values (logCPM) after removing the effects of the surrogate variables to cluster the (**A**) articular cartilage samples; and (**B**) subchondral bone samples to get an overview of similarities and differences between samples. Each samples’ age and location are indicated by point shape and color, respectively. Locations are F = fetlock (metatarsophalangeal joint), H = hock (tarsocrural joint), and S = stifle (femoropatellar joint).

For AC (Figure 1A), Dimension 1 separates the adults from the foals and explains 47.9% of the total variability in gene expression values. Dimension 2 explains 8.6% of the total variability and roughly separates foals by location, but not adults. Similarly, for SCB (Figure 1B), Dimension 1 separates the adults and foals and explains 66.8% of the total variability in gene expression values. Dimension 2 explains 4.8% of the total variability and separates foals by location, particularly the femoropatellar joint (stifle) from the other two. Adults were not expected to cluster by location in either tissue due to the four pooled samples. 

### 3.2. Differential Expression

#### 3.2.1. Articular Cartilage

**Figure 2:**
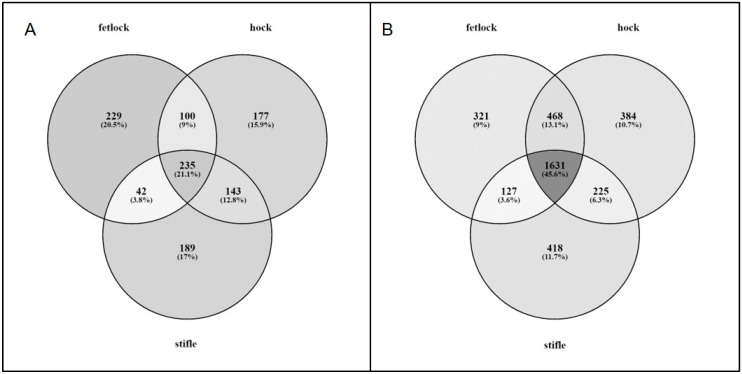
Venn diagrams showing overlap between differentially expressed (DE) genes in the three joint locations in (**A**) articular cartilage (AC) and (**B**) subchondral bone (SCB). Percentages are in reference to the total number of unique DE genes in each tissue (1115 genes in AC, 3574 in SCB).

In AC, 1115 genes were differentially expressed (DE) (FC > |1.5|, global FDR < 0.05) between adults and foals; 642 genes were upregulated and 446 downregulated in the foal samples when compared to the adults (Table 1; Table S2). Genes that were DE in each of the three joint locations were not completely concordant (Figure 2A). The gene set comprising the union of DE genes across all 3 locations was used in downstream analysis to characterize the pathways differentially expressed between foal and adult AC.

#### 3.2.2. Subchondral Bone

In SCB, 3574 genes were DE (FC > |1.5|, global FDR < 0.05) between adults and foals; 1658 genes were upregulated and 1896 downregulated in the foal samples when compared to the adults (Table 1; Table S3). Genes that were DE in each of the three joint locations were not completely concordant (Figure 2B). The gene set comprising the union of DE genes across all 3 locations was used in downstream analysis to characterize the pathways differentially expressed between foal and adult SCB.

### 3.3. Functional Annotation and Pathway Analysis

#### 3.3.1. Articular Cartilage

PANTHER reports a hierarchical organization of GO-Slim terms that are overrepresented in the genes differentially expressed between foal and adult tissue. GO terms could be assigned to 965 of the 1115 unique DE genes found in AC. Among these, 54 Biological Process terms were overrepresented, falling within 17 hierarchal categories; 23 Molecular Function terms were overrepresented, falling within 10 hierarchal categories; and 28 Cellular Component terms were overrepresented, falling within 11 hierarchal categories. Some genes were included under more than one GO-Slim term. The terminal hierarchical overrepresented terms are shown in Table 2. 

PANTHER identified 18 enriched pathways (9 hierarchal categories) containing 965 genes DE between foal and adult AC. Some genes fell within more than one pathway. In contrast, Reactome assigned 557 of the 965 DE genes to 1097 pathways, of which 11 reached the designated level of statistical significance (FDR < 0.05). Eleven pathways were identified as enriched by both programs: extracellular matrix (ECM) organization, collagen chain trimerization, collagen formation, collagen biosynthesis and modifying enzymes, assembly of collagen fibrils and other multimeric structures, collagen degradation, degradation of the ECM, ECM proteoglycans, integrin cell surface interactions, NCAM1 interactions, and NCAM signaling for neurite outgrowth (Table 3). 

When considering the pathways containing genes differentially expressed between foal and adult AC that were identified as enriched by both PANTHER and Reactome, two broad categories emerged: (1) pathways involved in the production and turnover of the extracellular matrix (ECM organization, collagen chain trimerization, collagen biosynthesis and modifying enzymes, collagen formation, degradation of the ECM, collagen degradation, ECM proteoglycans, assembly of collagen fibrils and other multimeric structures, integrin cell surface interactions); and (2) pathways involved in neural development (NCAM1 interactions, NCAM signaling for neurite out-growth). These functional groupings were also reflected in the overrepresented GO-Slim terms. Enriched pathways (and GO-Slim terms) relevant to ECM organization were largely driven by upregulation of numerous collagen sub-types in the foals, as well as associated matrix metalloproteinases (MMPs), and ADAMTS (a disintegrin and metalloproteinase with thrombospondin motifs) protein family members. There was significant overlap of genes across these pathways (Table 4; Table S5). Some collagen subtypes were also prominent members of the pathways more related to neural development, as were cell adhesion molecules. Again, significant overlap of genes between pathways was observed (Table 4; Table S5).

Additional pathways related to neural development and neurotransmission were identified as enriched by PANTHER, but not by Reactome (Neurotransmitter receptors and postsynaptic signal transmission, Transmission across chemical synapses, Neuronal system, Axon guidance) (Table 3). This functional grouping was also reflected by several overrepresented GO-Slim terms, including dendritic spine organization, synapese assembly, regulation of axonogenesis, and positive regulation of synaptic transmission, among others (Table 2). Gene families identified within these pathways (and GO-Slim terms) included glutamate receptors (e.g., GRIN2D, GRIA3, GRIK3), semaphorins (e.g., SEMA3D, SEMA7A, SEMA4D), SH3 and multiple ankyrin repeat domains proteins (SHANK1, SHANK2, SHANK3), and potassium channels (e.g., KCNA1, KCNN3, KCNJ15). 

#### 3.3.2. Subchondral Bone

PANTHER reports a hierarchical organization of GO-Slim terms that are overrepresented in the genes differentially expressed between foal and adult tissue. GO terms could be assigned to 2923 of the 3574 unique DE genes found in SCB. Among these, 42 Biological Process terms were overrepresented, falling within 13 hierarchal categories; 27 Molecular Function terms were overrepresented, falling within 11 hierarchal categories; and 34 Cellular Component terms were overrepresented, falling within 12 hierarchal categories. Some genes were included under more than one GO-Slim term. The terminal hierarchical overrepresented terms are shown in Table 5.

PANTHER identified 79 enriched pathways (42 hierarchal categories) containing 2923 genes DE between foal and adult SCB. Some genes fell within more than one pathway. In contrast, Reactome assigned 1789 of the 2923 DE genes to 1769 pathways, of which eight reached the designated level of statistical significance (FDR < 0.05). Three additional pathways nearly reached significance (FDR *p* = 0.052 in the Reactome analysis. Eight pathways were identified as enriched by both programs: ECM organization, neutrophil degranulation, RHO GTPase effectors, signaling by RHO GTPases, amplification of signal from the kinetochores, amplification of signal from unattached kinetochores via a MAD2 inhibitory signal, and resolution of sister chromatid adhesion. The three pathways that nearly reached significance in the Reactome analysis were identified as enriched by PANTHER: deposition of new CENPA-containing nucleosomes at the centromere, nucleosome assembly, and defective B3GALTL causes Peters-plus syndrome (Table 6; Table S7). This last pathway is identical to the normal pathway, O-glycosylation of TSR domain-containing proteins (R-HSA-5173214). 

When considering the pathways containing genes differentially expressed between foal and adult SCB that were identified as enriched by both PANTHER and Reactome, two broad categories emerged: (1) pathways involved in the cell cycle and signal transduction (resolution of sister chromatid cohesion, amplification of signal from kinetochores, amplification of signal from unattached kinetochores via a MAD2 inhibitory signal, deposition of new CENPA-containing nucleosomes at the centromere, nucleosome assembly, RHO GTPase effectors, signaling by RHO GTPases); and (2) pathways involved in ECM organization and tissue formation (ECM organization, integrin cell surface interactions, O-glycosylation of TSR domain-containing proteins). The neutropil degranulation pathway, which was the top pathway in both analyses, does not fall within either of these categories. There was substantial overlap of genes between pathways falling under the same broad category, but little overlap between pathways in different categories (Table S8). Interestingly, the vast majority of genes found in the cell cycle/signal transduction and neutrophil degranulation pathways were upregulated in foals, while those found in pathways involved in ECM organization were more evenly split between upregulation and downregulation (Table S8). In contrast to AC, in which only 60 DE genes comprised the enriched pathways (5% of all DE genes in this tissue), 402 DE genes were found within the enriched SCB pathways (11% of all DE genes in this tissue).

Gene families prominent in the cell cycle/signal transduction pathways included Rho GTPase activating proteins, Rho guanine nucleotide exchange proteins, actin related proteins, centromere proteins, histones, kinesin family members, ras homolog family members, and tubulins. The ECM organization/tissue formation pathways were dominated by ADAM/ADAMTS family members, integrins, and collagens and other ECM molecules (e.g., aggrecan, versican). The neutrophil degranulation pathway was not dominated by any particular protein families other than the expected cell surface glycoproteins (e.g., CD33, CD47, CD53). Evaluation of overrepresented protein classes among the 164 DE genes in this pathway using PANTHER revealed a mix of receptors, hydrolases, signaling molecules, enzyme modulators, peroxidases, and defense/immunity proteins. 

## 4. Discussion

Here, we report differential expression of genes in healthy AC and SCB in neonatal foals and adult horses. To our knowledge, this is the first time that a comparison of joint tissue-specific gene expression between life stages has been reported in this species. Functional annotation and pathway analysis provide a framework within to evaluate this expression data and help identify putative candidate pathways and genes that may play a role in skeletogenesis. It is not surprising that the majority of both enriched pathways and overrepresented gene ontology terms found in our data reflect a state of growth, high metabolic activity, and tissue turnover in neonatal foals. However, the identification of overrepresented biological process and pathways related to neural development and neurotransmission in the AC and related to innate immunity in the SCB was unexpected. Further exploration of these pathways may enhance our understanding of skeletal development, particularly in the post-natal period, as well as elucidate novel potential mechanisms for orthopedic disease in the horse. 

Growth and maturation of the long bones occurs via endochondral ossification, a process that is regulated by the interactions of a number of hormones, growth factors, transcription factors, and extracellular matrix molecules secreted by chondrocytes [1,3]. At the ends of long bones, endochondral ossification within the articular-epiphyseal cartilage complex (a secondary center of ossification) results in transformation of the epiphyseal growth cartilage into mature subchondral bone capped by a thin layer of articular cartilage at the joint surface [42]. While chondrocytes are considered the central drivers of endochondral ossification, osteoblasts and osteoclasts also play a distinct and critical role in the process [1,2,3]. Experimentation, particularly in mouse models, has elucidated many complex interactions between these cells and has identified a number of signaling pathways that can lead to prenatal or postnatal disruption of normal skeletogenesis [43,44,45,46,47], although there is still much that is not known, particularly about chondrocyte physiology [48]. Key signaling molecules in endochondral ossification that have been identified across species include insulin-like growth factors (IGF), Indian hedgehog (IHH), bone morphogenetic proteins (BMPs), fibroblastic growth factors (FGF), cyclins, and Wingless (WNT) and Notch signaling factors [3,22,49,50]. Indeed, many of the DE genes identified in our data play a role in these signaling pathways. In some cases, signaling factors play opposite roles under different circumstances, depending on concentration, microenvironment, or stage of development [3,51,52]. 

Mutations within transcription factors and other signaling molecules that regulate endochondral ossification, as well as mutations in genes encoding major ECM components, have been associated with global skeletal dysplasias [22]. However, delayed or disturbed endochondral ossification is also associated with focal abnormalities, which can manifest as osteochondrosis (OC), a form of DOD frequently diagnosed in horses as well as a number of other species including humans, pigs, dogs, cattle, and chickens [53]. Numerous studies have attempted to identify key risk genes OC by performing differential gene expression analysis in diseased (or “predisposed”) tissue compared to normal tissue from foals. The majority of these have been targeted studies utilizing quantitative polymerase chain reaction (qPCR) to investigate specific genes or pathways known to play a role in endochondral ossification [54,55,56,57,58]. These include various types of collagen and extracellular matrix components [55,58] and multiple members of the Wnt/β-catenin signaling pathway [54], many of which, not surprisingly, we also identified as DE in AC and/or SCB between foals and adults. Relatively few reports record the use of a less focused gene expression-based approach for identifying putative candidate genes for OC, but they all identified DE genes that had not previously been recognized as potentially playing a role in disease pathogenesis—and indeed, some that had never previously been reported in cartilage [59,60,61]. This emphasizes the power of a more global approach when investigating complex disease. Although our work was done in normal tissue, our RNAseq approach identified several novel pathways that have not previously been directly implicated in endochondral ossification.

In addition to finding expected differences in genes/pathways involved in extracellular matrix formation and organization in the AC, we also identified enriched pathways related to neural development and neurotransmission. Examination of the specific DE genes driving this enrichment revealed that many fell within the glutamate signaling pathway. Although glutamate is primarily thought of as a neurotransmitter, there is a growing body of evidence supporting its role as a signaling molecule (autocrine and/or paracrine) in cartilage and bone [62,63,64]. Further, although mature AC is not innervated, peripheral nerve fibers and neurotransmitters are known to play important roles in endochondral ossification, via effects on vascularization and proliferation and differentiation of progenitor cells [65]. The two specific pathways for neural development that were identified as enriched in AC related to the actions of the gene *NCAM1* (neural cell adhesion molecule). This gene encodes a transmembrane protein that is involved in cell-cell and cell-ECM binding. Although *NCAM1* has primarily been associated with neurons, a recent study reported it to be a predictive marker for chondrogenic potential of mesenchymal stem cells [66], suggesting that it may also play an important primary role in the development of musculoskeletal tissues. Glutamate and other neurotransmitters have been linked to inflammatory/degenerative joint pathology [65,67], but to our knowledge have not been examined for a potential role in DOD.

In the subchondral bone, we identified enriched pathways related to innate immunity, specifically the neutrophil degranulation pathway, which was significant in both PANTHER and Reactome analyses. Immune cells, including both neutrophils and lymphocytes, are known to play an important role in bone remodeling, primarily via effects on bone resorption [68]. However, neutrophils have also been shown to enhance osteogenesis in triple cell co-culture models in vitro [69]. The presence of neutrophils resulted in increased mineralization, increased production of markers of osteogenesis, and upregulation of numerous genes that play a role in osteogenesis, including extracellular matrix proteins, bone morphogenetic proteins, and colony stimulating factors, among others [69]. In our data set, of the 165 DE genes falling within the neutrophil degranulation pathway, 144 (87.3%) were upregulated in the foals compared to the adult, consistent with a putative role for this pathway in normal skeletal development. Notably, although only the neutrophil degranulation pathway was significantly enriched in our analyses, many of the DE genes falling within this pathway play roles in other aspects of the immune system, including complement activation, interleukin signaling, and toll-like receptor signaling. Each of these has been reported to play a role in osteogenesis, although most studies have focused on their putative role in disease and fracture repair [51,70,71,72,73,74,75], and relatively little seems to be known about their role in normal skeletal development. A role for immune response in the pathogenesis of OC has previously been suggested in pigs, based on global gene expression profiling (via microarray) in OC-affected compared to OC-unaffected articular cartilage [76]. However, this study did not investigate gene expression in subchondral bone.

Limitations of this study include the relatively small number of animals in each group and the age range of the foals. It is possible that subtle differences in gene expression between 1 day and 4 weeks of age (the range of our samples) could have affected some of our findings. However, the clear separation between foal and adult samples in both our raw and surrogate variable-corrected MDS plots suggest that this is unlikely. Evaluation of differences in gene expression between foals at different time points during the early postnatal period would be a valuable area of future study, particularly if comparisons with fetal tissue samples could also be made. This could help to identify transient changes in gene expression during the time when endochondral ossification is ongoing and pathology develops. Additionally, while all tissue samples were free of macroscopic abnormalities, histopathology was not performed and therefore subclinical microscopic disease cannot be ruled out. Finally, there are inherent limitations with functional gene annotation and pathway analysis. Pathway annotations are often based largely on text-mining of the literature, so are restricted by the context in which gene-gene interactions have been reported. Further, many DE genes fall within multiple GO terms and pathways, which can complicate the interpretation of results. Thus, it is possible that some of the specific overrepresented GO terms and statistically enriched pathways found in our data were identified by chance, although our data were consistent with other previously reported experimental findings. Validation of tissue gene expression via qPCR as well as analyses to determine if expression differences are translated into differences in protein production would be important next steps towards determining the mechanistic impact of our findings. 

## 5. Conclusions

Differential expression analysis and functional annotation of genes expressed in normal articular cartilage and subchondral bone of neonatal and adult horses reveals numerous pathways that that may be involved in post-natal skeletal development and growth and could be considered candidates for derangements in the development of orthopedic disease in young animals. Although some of these pathways, such as those related to extracellular matrix production and turnover, might be considered intuitively obvious, others, such as those related to neurotransmission (glutamate signaling) and innate immunity (neutrophil degranulation) are novel and represent a new avenue to explore as we seek to elucidate the complexities of skeletogenesis. In future work, these data can be combined with genome-wide association study (GWAS) and quantitative trait locus (QTL) results [76,77] and/or with gene expression data from diseased tissue to prioritize specific putative candidate genes involved in disease pathogenesis in the horse. 

## Figures and Tables

**Table 1:** Number of DE genes with FC > |1.5| and global FDR *p* < 0.05 in articular cartilage and subchondral bone. DE is calculated by joint location. F = fetlock (metatarsophalangeal joint); H = hock (tarsocrural joint); S = stifle (femoropatellar joint).

		Foal.F vs. Adult.F	Foal.H vs. Adult.H	Foal.S vs. Adult.S
Articular Cartilage	Down	212	288	260
	Not Significant	15,832	15,785	15,831
	Up	396	367	349
Subchondral Bone	Down	1343	1360	1250
	Not Significant	15,461	15,296	15,606
	Up	1205	1353	1153

**Table 2:** Overrepresented GO-Slim terms among differentially expressed genes in AC. The reference list is Homo sapiens UniProt IDs and includes 20996 genes; the analyzed list is comprised of the UniProt IDs for 965 DE genes identified from AC samples. FDR = false discovery rate (significance set at 0.05). A complete hierarchical list of overrepresented terms can be found in Table S4.

	Genes in Reference List	Genes in Analyzed List	Fold Enrichment	Raw *p*-Value	FDR
GO-Slim Biological Process					
extracellular matrix organization (GO:0030198)	69	16	5.05	7.76 × 10^−7^	0.0002
microtubule-based movement (GO:0007018)	87	15	3.75	3.85 × 10^−5^	0.0041
regulation of axonogenesis (GO:0050770)	27	8	6.45	1.10 × 10^−4^	0.007
chemical synaptic transmission (GO:0007268)	330	33	2.18	8.10 × 10^−5^	0.0073
postitive regulation of synaptic transmission (GO:0050806)	24	7	6.35	3.22 × 10^−4^	0.017
cell adhesion (GO:0007155)	373	34	1.98	3.39 × 10^−4^	0.017
Wnt signaling pathway (GO:0016055)	98	14	3.11	3.98 × 10^−4^	0.018
positive regulation of signal transduction (GO:0009967)	112	15	2.91	4.65 × 10^−4^	0.018
tissue development (GO:0009888)	117	15	2.79	7.01 × 10^−4^	0.026
angiogenesis (GO:0001525)	30	7	5.08	1.00 × 10^−3^	0.035
regulation of chemotaxis (GO:0050920)	22	6	5.93	1.16 × 10^−3^	0.039
axonogenesis (GO:0007409)	98	13	2.89	1.18 × 10^−3^	0.039
amino acid transport (GO:0007416)	15	5	7.25	1.45 × 10^−3^	0.044
Notch signaling pathway (GO:0007219)	32	7	4.76	1.39 × 10^−3^	0.044
dendritic spine organization (GO:0097061)	3	3	21.76	1.53 × 10^−3^	0.045
synapse assembly (GO:0007416)	15	5	7.25	1.45 × 10^−3^	0.045
negative regulation of neurogenesis (GO:0050768)	24	6	5.44	1.70 × 10^−3^	0.048
GO-Slim Molecular Function					
extracellular matrix structural component (GO:0005201)	53	17	6.98	6.46 × 10^−9^	3.26 × 10^−6^
microtubule binding (GO:0008017)	123	17	3.01	1.43 × 10^−4^	0.01
transmembrane receptor protein kinase activity (GO:0019199)	62	10	3.51	1.16 × 10^−3^	0.031
cation transmembrane transporter activity (GO:0008324)	449	37	1.79	1.10 × 10^−3^	0.031
metallopeptidase activity (GO:0008237)	103	14	2.96	6.23 × 10^−4^	0.035
calcium ion binding (GO:0005509)	205	21	2.23	1.46 × 10^−3^	0.037
cadherin binding (GO:0045296)	43	8	4.05	1.60 × 10^−3^	0.039
peptidase inhibitor activity (GO:0030414)	117	14	2.6	1.88 × 10^−3^	0.043
ATP-dependent microtubule motor activity, plus-end-directed (GO:0008574)	25	6	5.22	2.03 × 10^−3^	0.045
gated channel activity (GO:0022836)	160	17	2.31	2.45 × 10^−3^	0.046
GO-Slim Cellular Component					
collagen trimer (GO:0005581)	10	6	13.05	3.88 × 10^−5^	0.0022
microtubule associated complex (GO:0005875)	58	12	4.5	4.89 × 10^−5^	0.0022
extrinsic component of plasma membrane (GO:0019897)	28	8	6.22	1.36 × 10^−4^	0.0041
cell-cell junction (GO: 0005911)	70	12	3.73	2.35 × 10^−4^	0.0066
cation channel complex (GO:0034703)	94	14	3.24	2.72 × 10^−4^	0.0072
microtubule (GO:0005874)	162	19	2.55	4.04 × 10^−4^	0.0095
postsynaptic density (GO:0014069)	46	9	4.26	6.06 × 10^−4^	0.013
postsynaptic membrane (GO:0045211)	58	10	3.75	7.35 × 10^−4^	0.015
ionotropic glutamate receptor complex (GO:0008328)	33	7	4.62	1.62 × 10^−3^	0.029
extracellular space (GO:0005615)	863	61	1.54	1.73 × 10^−3^	0.029
basement membrane (GO:0005604)	4	3	16.32	2.59 × 10^−3^	0.036

**Table 3:** Results from pathway analysis in PANTHER and Reactome. Adjacent pathways sharing the same background color are hierarchically related to each other in the PANTHER analysis. Fold-enrichment is not reported by Reactome (NA = not applicable). FDR = false discovery rate; ECM = extracellular matrix; R-HSA-XXX = Reactome pathway identifiers.

Pathway	Genes in Reference List	Genes in Analyzed List	Fold Enrichment	Raw *p*-Value	FDR
PANTHER					
Collagen chain trimerization (R-HSA-8948216)	44	14	6.92	1.52 × 10^−7^	4.15 × 10^−5^
Collagen biosynthesis and modifying enzymes (R-HSA-1650814)	67	18	5.85	2.38 × 10^−8^	8.68 × 10^−6^
Collagen formation (R-HSA-1474290)	89	21	5.13	1.17 × 10^−8^	8.53 × 10^−6^
ECM organization (R-HSA-1474244)	299	54	3.93	1.02 × 10^−15^	2.25 × 10^−12^
NCAM1 interactions (R-HSA-8948216)	42	13	6.73	5.48 × 10^−7^	0.0001
NCAM signaling for neurite outgrowth (R-HSA-375165)	60	14	5.08	3.54 × 10^−6^	0.00055
Axon guidance (R-HSA-422475)	550	52	2.06	3.97 × 10^−6^	0.00058
Collagen degradation (R-HSA-1442490)	64	16	5.44	3.27 × 10^−7^	7.16 × 10^−5^
Degradation of the ECM (R-HSA-1474228)	140	26	4.04	1.73 × 10^−8^	9.48 × 10^−6^
Assembly of collagen fibrils and other multimeric structures (R-HSA-2022090)	60	15	5.44	7.60 × 10^−7^	0.00013
Integrin cell surface interactions (R-HSA-216083)	84	20	5.18	2.28 × 10^−8^	9.99 × 10^−6^
ECM proteoglycans (R-HSA-3000178)	76	17	4.87	5.46 × 10^−7^	0.00011
Kinesins (R-HSA-983189)	61	13	4.64	1.82 × 10^−5^	0.0025
Hemostasis (R-HSA-109582)	671	54	1.75	1.99 × 10^−4^	0.021
Elastic fibril formation (R-HSA-1566948)	45	9	4.35	5.27 × 10^−4^	0.046
Neurotransmitter receptors and postsynaptic signal transmission (R-HSA-112314)	148	19	2.79	1.43 × 10^−4^	0.017
Transmission across chemical synapses (R-HSA-112315)	218	26	2.59	4.57 × 10^−5^	0.0056
Neuronal system (R-HSA-112316)	361	42	2.53	2.29 × 10^−7^	5.57 × 10^−5^
Reactome					
ECM organization (R-HSA-1474244)	329	54	NA	1.26 × 10^−8^	1.49 × 10^−5^
Collagen chain trimerization (R-HSA-8948216)	44	14		3.95 × 10^−6^	0.0017
Integrin cell surface interactions (R-HSA-216083)	86	20		4.40 × 10^−6^	0.0017
Collagen biosynthesis and modifying enzymes (R-HSA-1650814)	76	18		1.02 × 10^−5^	0.003
NCAM1 interactions (R-HSA-419037)	44	13		1.86 × 10^−5^	0.0041
Collagen formation (R-HSA-1474290)	104	21		2.07 × 10^−5^	0.0041
Degradation of the ECM (R-HSA-1474228)	148	26		2.45 × 10^−5^	0.0041
Collagen degradation (R-HSA-1442490)	69	16		4.00 × 10^−5^	0.0059
ECM proteoglycans (R-HSA-3000178)	79	17		5.75 × 10^−5^	0.0075
Assembly of collagen fibrils and other multimeric structures (R-HSA-2022090)	67	15		1.00 × 10^−4^	0.012
NCAM signaling for neurite out-growth (R-HSA-375165)	69	14		4.46 × 10^−4^	0.048

**Table 4:** Genes found within enriched pathways identified by both PANTHER and Reactome based on DE in articular cartilage. Only 3 out of 60 genes were found in a single pathway, while 8 genes (all collagen subtypes) were found in all enriched pathways. For each gene “x” designates the pathway(s) in which it was found.

Gene Family	Gene Symbol(s)	ECM Organization	Collagen Chain Trimerization	Collagen Biosynthesis and Modifying Enzymes	Collagen Formation	Degradation of the ECM	Collagen Degradation	ECM Proteoglycans	Assembly of Collagen Fibrils and Other Multimeric Structures	Integrin Cell Surface Interactions	NCAM1 Interactions	NCAM Signaling for Neurite Outgrowth
Upregulated in foals												
ADAM metallopeptidase domain	ADAM12 ADAM19	x										
ADAM metallopeptidase with thrombospondin	ADAMTS2 ADAMTS3	x		x	x							
	ADAMTS8	x				x						
Calcium voltage-gated channel subunit	CACNA1G CACNA1H										x	x
Calpain	CAPN6	x				x						
Collagen	COL2A1 COL4A1 COL9A1 COL9A2 COL9A3	x	x	x	x	x	x	x	x	x	x	x
	COL8A1	x	x	x	x	x	x		x	x		
	COL11A1 COL11A2	x	x	x	x	x			x			
	COL16A1	x	x	x	x	x	x			x		
	COL18A1	x	x	x	x	x	x		x	x		
	COL21A1	x	x	x	x							
Elastin	ELN	x				x						
Immunoglobulin superfamily	F11R	x								x		
	NCAM1	x						x			x	x
Fibulin	FBLN2	x										
Fibrillin	FBN2	x				x						
Hyaluronan and proteoglycan link protein	HAPLN1	x						x				
Heparain sulfate proteoglycan	HSPG2	x				x		x	x			
Integrin subunit	ITGA10	x								x		
VEGF receptor	KDR	x								x		
Lysyl oxidase	LOXL2	x							x			
Matrilin	MATN1 MATN3	x						x				
Microfibrillar associated protein	MFAP2	x										
Matrix metalloproteinase	MMP2 MMP15	x				x	x					
	MMP9	x			x	x	x		x			
Nidogen	NID2	x										
Platelet and endothelial cell adhesion molecule	PECAM1	x								x		
Serpin	SERPINH1	x		x	x							
Secreted protein acidic and cysteine rich	SPARC	x						x				
Spectrin	SPTBN2											x
Downregulated in foals												
Calpain	CAPN2	x				x						
Transmembrane 4 superfamily	CD151	x			x				x			
Collagen	COL4A3	x	x	x	x	x	x	x	x	x	x	x
	COL6A5 COL6A6	x	x	x	x	x	x	x	x	x	x	x
Small leucine-rich proteoglycan	DCN	x				x		x				
Integrin subunit	ITGA3 ITGAM	x								x		
	ITGB5	x						x		x		
latent TGFb binding protein	LTB2 LTB3 LTB4	x										
Procollagen C-endopeptidase enhancer	PCOLCE2	x		x	x							
Prion protein	PRNP										x	x
Signal peptide, CUB domain and EGF-like domain	SCUBE1	x				x						
Secreted phosphoprotein	SPP1	x				x				x		
Sialyltransferase	ST8SIA4										x	x
Tissue inhibitor of metalloproteinase	TIMP2	x				x						
Tenascin	TNXB	x						x				

**Table 5:** Overrepresented GO-Slim terms among DE genes in SCB. The reference list is Homo sapiens UniProt IDs and includes 20996 genes; the analyzed list is comprised of the UniProt IDs for 2923 DE genes identified from SCB samples. FDR = false discovery rate (significance set at 0.05). A complete hierarchical list of overrepresented terms can be found in Table S6.

	Genes in Reference List	Genes in Analyzed List	Fold Enrichment	Raw *p*-Value	FDR
GO-Slim Biological Process					
mitotic nuclear division (GO:0140014)	209	63	2.17	5.19 × 10^−7^	8.47 × 10^−5^
positive regulation of cytosolic calcium ion concentration (GO:0007204)	65	24	2.65	1.33 × 10^−4^	0.0096
inflammatory response (GO:0006954)	101	32	2.28	1.50 × 10^−4^	0.01
intracellular signal transduction (GO:0035556)	777	151	1.4	2.24 × 10^−4^	0.013
extracellular matrix organization (GO:0030198)	69	24	2.5	3.32 × 10^−4^	0.018
tissue development (GO:0009888)	117	34	2.09	3.93 × 10^−4^	0.02
cytoskeleton organization (GO:0007010)	226	55	1.75	4.47 × 10^−4^	0.021
regulation of cell proliferation (GO:0042127)	96	29	2.17	5.34 × 10^−4^	0.022
transmembrane receptor protein tyrosine kinase signaling pathway (GO:0007169)	197	49	1.79	5.51 × 10^−4^	0.022
cell migration (GO:0016477)	177	45	1.83	6.07 × 10^−4^	0.024
neuron development (GO:0048666)	172	44	1.84	7.42 × 10^−4^	0.028
cell-cell adhesion (GO:0098609)	104	30	2.07	8.37 × 10^−4^	0.03
regulation of mitotic nuclear division (GO:0007088)	26	12	3.32	1.37 × 10^−3^	0.044
GO-Slim Molecular Function					
extracellular matrix structural component (GO:0005201)	53	22	2.98	3.00 × 10^−5^	0.0034
oxidoreductase activity (GO:0016491)	527	111	1.51	1.08 × 10^−4^	0.0055
cytokine receptor activity (GO:0004896)	69	25	2.6	1.81 × 10^−4^	0.0076
G-protein coupled peptide receptor activity (GO:0008528)	76	25	2.36	5.99 × 10^−4^	0.014
calcium ion binding (GO:0005509)	205	51	1.79	4.92 × 10^−4^	0.014
growth factor binding (GO:0019838)	46	18	2.81	7.18 × 10^−4^	0.017
actin filament binding (GO:0051015)	65	21	2.32	1.49 × 10^−3^	0.029
transmembrane receptor protein kinase activity (GO:0004714)	60	20	2.39	1.60 × 10^−3^	0.03
peptidase inhibitor activity (GO:0030414)	117	32	1.96	1.48 × 10^−3^	0.03
C-C chemokine binding (GO:0019957)	24	11	3.29	2.25 × 10^−3^	0.038
metallopeptidase activity (GO:0008237)	103	28	1.95	2.91 × 10^−3^	0.043
GO-Slim Cellular Component					
integral component of plasma membrane (GO:0005887)	733	165	1.62	5.71 × 10^−8^	1.28 × 10^−5^
collagen-containing ECM (GO:0062023)	33	15	3.27	4.09 × 10^−4^	0.0087
receptor complex (GO:0043235)	177	45	1.83	6.07 × 10^−4^	0.011
focal adhesion (GO:0005925)	25	12	3.45	1.05 × 10^−3^	0.016
condensed nuclear chromosome kinetochore (GO:0000778)	9	7	5.59	1.69 × 10^−3^	0.022
microtubule (GO:0005874)	162	40	1.77	2.30 × 10^−3^	0.025
extracellular space (GO:0005615)	863	157	1.31	2.06 × 10^−3^	0.025
cyclin-dependent protein kinase holoenzyme complex (GO:0000307)	28	12	3.08	2.24 × 10^−3^	0.026
spindle (GO:0005819)	46	16	2.5	2.91 × 10^−3^	0.03
cell surface (GO:0009986)	322	67	1.49	3.75 × 10^−3^	0.038

**Table 6:** Results from pathway analysis in PANTHER and Reactome. Only the top 25 (by FDR) terminal hierarchical pathways from the PANTHER analysis are listed here; the complete hierarchical list of enriched pathways can be found in Table S7**.** Fold-enrichment is not reported by Reactome (NA = not applicable). FDR = false discovery rate; ECM = extracellular matrix; R-HSA-XXX = Reactome pathway identifiers.

Pathway	Genes in Reference List	Genes in Analyzed List	Fold Enrichment	Raw *p*-Value	FDR
PANTHER					
Neutrophil degranulation (R-HSA-6798695)	479	167	2.5	1.2 × 10^−21^	1.31 × 10^−18^
Resolution of sister chromatid cohesion (R-HSA-2500257)	122	50	2.94	2.69 × 10^−9^	4.54 × 10^−7^
RHO GTPases activate formins (R-HSA-5663220)	135	51	2.71	2.52 × 10^−8^	3.25 × 10^−6^
Amplification of signal from unattached kinetochores via a MAD2 inhibitory signal (R-HSA-141444)	92	39	3.04	1.05 × 10^−7^	1.15 × 10^−5^
Integrin cell surface interactions (R-HSA-216083)	84	36	3.08	1.73 × 10^−7^	1.65 × 10^−5^
ECM proteoglycans (R-HSA-3000178)	76	31	2.93	3.60 × 10^−6^	2.46 × 10^−4^
Deposition of new CENPA-containing nucleosomes at the centromere (R-HSA-606279)	54	25	3.33	4.40 × 10^−6^	3.60 × 10^−4^
Non-integrin membrane-ECM interactions (R-HSA-3000171)	59	26	3.17	5.76 × 10^−6^	3.60 × 10^−4^
Defective B3GALTL causes Peters-plus syndrome (PpS) (R-HSA-5083635)	38	20	3.78	9.26 × 10^−6^	5.63 × 10^−4^
O-glycosylation of TSR domain-containing proteins (R-HSA-5173214)	39	20	3.68	1.24 × 10^−5^	7.35 × 10^−4^
Collagen degradation (R-HSA-1442490)	64	25	2.81	5.24 × 10^−5^	0.0026
Assembly of collagen fibrils and other multimeric structures (R-HSA-2022090)	60	24	2.87	6.26 × 10^−5^	0.0029
Kinesins (R-HSA-983189)	61	24	2.83	7.11 × 10^−5^	0.0033
NCAM1 interactions (R-HSA-419037)	42	19	3.25	7.67 × 10^−5^	0.033
Molecules associated with elastic fibres (R-HSA-2129379)	38	18	3.4	7.43 × 10^−5^	0.0033
Separation of sister chromatids (R-HSA-2467813)	186	50	1.93	8.50 × 10^−5^	0.0035
GPVI-mediated activation cascade (R-HSA-114604)	35	17	3.49	9.21 × 10^−5^	0.0037
Platelet degranuation (R-HSA-114608)	127	38	2.15	1.04 × 10^−4^	0.0039
Cell surface interactions at the vascular wall (R-HSA-202733)	198	50	1.81	3.96 × 10^−4^	0.012
Laminin interactions (R-HSA-3000157)	30	14	3.35	5.15 × 10^−4^	0.015
Constituative signaling by aberrant PI3K in cancer (R-HSA-2219530)	55	20	2.61	5.72 × 10^−4^	0.016
Metabolism of folate and pterines (R-HSA-196757)	16	10	4.49	6.05 × 10^−4^	0.016
MyD88 deficiency (TLR2/4) (R-HSA-5602498)	10	8	5.75	6.91 × 10^−4^	0.018
Neuronal system (R-HSA-112316)	361	78	1.55	6.78 × 10^−4^	0.018
Signaling by interleukins (R-HSA-449147)	449	93	1.49	6.88 × 10^−4^	0.018
Reactome					
Neutrophil degranulation (R-HSA-6798695)	480	164	NA	1.47 × 10^−9^	2.79 × 10^−6^
RHO GTPase effectors (R-HSA-195258)	326	105		1.49 × 10^−5^	0.014
ECM organization (R-HSA-1474244)	329	105		2.13 × 10^−5^	0.014
Signaling by RHO GTPases (R-HSA-194315)	457	136		3.79 × 10^−5^	0.018
Aplification of signal from the kinetochores (R-HSA-141424)	94	39		6.82 × 10^−5^	0.022
Amplification of signal from unattached kinetochores via a MAD2 inhibitory signal (R-HSA-141444)	94	39		6.82 × 10^−5^	0.022
Resolution of sister chromatid cohesion (R-HSA-2500257)	134	50		9.86 × 10^−5^	0.025
Integrin cell surface interactions (R-HSA-216083)	86	36		1.07 × 10^−4^	0.025
Deposition of new CENPA-containing nucleosomes at the centromere (R-HSA-606279)	54	25		2.71 × 10^−4^	0.052
Nucleosome assembly (R-HSA-774815)	54	25		2.71 × 10^−4^	0.052
Defective B3GALTL causes Peters-plus syndrome (PpS) (R-HSA-5083635)	39	20		3.04 × 10^−4^	0.052

## Supplementary Materials

The following are available online at https://www.mdpi.com/2073-4425/10/10/745/s1, Figure S1: Percentage of reads mapped to transcriptome by Salmon for SBC and AC; Figure S2: TMM normalization factors for SCB and AC; Figure S3: MDS plot of AC and SCB gene expression data prior to the removal of surrogate variables; Figure S4: Boxplots of the estimated surrogate variable (SV) values for AC; Figure S5: Boxplots of the estimated surrogate variable (SV) values for SCB. Table S1: Description of samples; Table S2: Genes found to be differentially expressed in AC with FC > |1.5|; Table S3: Genes found to be differentially expressed in SCB with FC > |1.5|; Table S4: Hierarchical listing of overrepresented GO terms in AC; Table S5: Genes falling within pathways identified as enriched in AC; Table S6: Hierarchical listing of overrepresented GO terms in SCB; Table S7: Hierarchical listing of pathways identified by PANTHER analysis as enriched in SCB; Table S8: Genes falling within pathways identified as enriched in SCB.

## Appendix A

This appendix contains more detailed methods for quality control and analysis of RNAseq data, to augment information provided in Materials and Methods.

### Appendix A.1. Quality Control

Quality control on all 112 fastq files (from 56 samples) was performed using fastp [24] (version 0.19.5) and summarized using MultiQC [78] (version 1.6). The adult samples sequenced at UMGC had average per-base read quality scores dropping below 30 starting around 80 bases. Therefore, all fastq files were trimmed using Trimmomatic [25] (version 0.38) in paired end mode to trim any remaining adaptors, then trim leading or trailing bases with quality scores below 28, then discarding a read pair if either read was shorter than 30 bases. After trimming, average per-base read quality scores were above 30 through the entire 100 bases for all retained reads. The UMGC samples lost ~3% of their paired-reads to trimming compared to the newer samples which only lost ~0.5% of their paired-reads.

### Appendix A.2. Alignment and Gene-Level Quantification

We used the EquCab3.0 genome assembly and NCBI’s Annotation Release 103. Salmon [26] (version 0.11.3) was used to quasi-map reads directly to the transcriptome and quantify the abundance of each transcript. The transcriptome was first indexed after discarding identical transcript sequences, then quasi-mapping in paired-end mode was performed to map reads to the transcriptome with additional arguments –seqBias and --gcBias to correct sequence-specific and GC content biases, respectively, and –numBootstraps=30 to compute bootstrap transcript abundance estimates. Gene-level counts were then estimated based on transcript level counts using the “bias corrected counts without an offset” method from the tximport package, which provides more accurate gene-level counts estimates and keeps multi-mapped reads in the analysis compared to traditional alignment-based methods [27]. At this point, gene counts for the two tissues were analyzed separately. Additionally, gene-level counts were added together for the two foals that had two samples of AC collected from the femoropatellar joint. For AC, percentage of reads mapped to the transcriptome ranged from 83.1–92.4%; read counts per sample ranged from 9.6–45.1 million. For SCB, percentage of mapped reads was 77.7–85.1%; read counts were 11–33.6 million per sample (Figure S1). Unmapped reads were discarded, retaining only mapped reads for statistical analysis.

### Appendix A.3. Normalization and Filtering

When comparing expression levels, the number of reads per gene need to be normalized not only because of the differences in total number of reads, but because there could be differences in RNA composition such that the total number of reads would not be expected to be the same. The TMM (trimmed mean of the M values) normalization [79] in the edgeR package [80] uses the assumption that most genes do not change to calculate a normalization factor for each sample to adjust for such biases in RNA composition. TMM normalization factors fluctuated between 0.7–1.29 and 0.77–1.27 in AC and SCB, respectively, and were generally above 1 in adult samples in both tissues (Figure S2), which suggests that there could be slight overall differences in RNA composition. While the NCBI EquCab3.0 Annotation Release 103 transcriptome has a total of 29,196 genes, a large proportion of these are not expected to have detectable expression. Therefore, the detection threshold was set at 1 cpm (counts per million) in at least 2 samples, which follows the recommendations by Chen et al. [28] for 10/(smallest library size in millions) cpm and smallest group replicate number. This filtering left 16,440 genes to be analyzed for differential expression in AC (accounting for 99.92% of reads) and 18,009 genes to be analyzed for differential expression in SCB (accounting for 99.91% of reads). After filtering, TMM normalization was performed again and normalized log2-based count per million values (logCPM) were calculated using edgeR’s ‘cpm’ function with prior.count=3 to help reduce variability in fold-changes of extremely low expression genes [28].

### Appendix A.4. Clustering

Multidimensional scaling (MDS) in the limma package [32] was used to check for outlier samples and any clustering structure in the raw sequence data. The top 5000 variable genes were chosen to construct the MDS plot. For AC (Figure S3), Dimension 1 separated the adults from the foals and explained 32% of the total variability in gene expression values. Dimension 2 explained 14% of the total variability and was a combination of foal age/individual horse (confounded) and sequencing year. Similarly, for SCB (Figure S3), Dimension 1 separated samples between adults and foals and explained 50% of the total variability in gene expression values. Dimension 2 explained 10% of the total variability and was again a combination of foal age/individual horse (confounded) and sequencing year. For both tissues, the samples did not distinctly cluster by joint location (i.e., metatarsophalangeal vs tarsocrural vs femoropatellar) through dimension 8. As noted in the main text, due to a lack of distinct joint differences, especially within adults, the 4 adult samples that were a mix of all three joint locations were assigned to one of the three locations for statistical analysis to balance out sex and replicate number. While this makes any within-location comparisons in adults unreliable, it still allows for valid foal vs. adult comparisons. Surrogate variable analysis (see below) was employed prior to differential expression testing. The MDS plots generated after surrogate variables were removed from the data are shown in Figure 1.

### Appendix A.5. Differential Expression Testing

The main variable of interest was age (foals vs adults), with location (metatarsophalangeal vs tarsocrural vs femoropatellar joints) of secondary interest, but there were many nuisance variables including individual horse effects, foal age, sequencing year, and sex. Some of the nuisance variables are partially or completely confounded, making it difficult to adjust for them in a traditional manner. Instead, we employed surrogate variables analysis [29,30] that separately estimated five continuous quantitative variables for each tissue that can be added as covariates to the model of age * location to correct for extraneous variation in the samples. We can also inspect the SV values to see which of our nuisance values they correspond to. For AC (Figure S4), the differences between 2012 and the other two years (MN vs. IL location) are corrected by sv2, while sv4 corrects for differences between 2016 and 2018. However, sv4 also separates most females from males, as sex and year are mostly confounded. Corrections for differences between individual horses in AC0 can be seen in sv1, sv3 and sv5. The surrogate variables for SCB (Figure S5) were slightly different. In this tissue, sv4 corrected for sequencing location (2012 vs 2016+2018) and while sv3 mostly separates 2016 from 2018, it seems to more strongly separate females and males. Corrections for differences between individual horses in SCB can be seen in sv1, sv2 and sv5.

Statistical corrections for the surrogate variables should be treated like any quantitative co-variate, like weight, by adding them to the statistical model. This allows the error associated with them to propagate correctly. The effects of surrogate variables can also be removed directly from the normalized logCPM values for visualization purposes, such as multidimensional scales (MDS) clustering (Figure 1) and heatmaps of expression patterns.
