# Supplementary material for: Differential Gene Expression in Articular Cartilage and Subchondral Bone of Neonatal and Adult Horses
Source: Genes (Basel). 2019 Sep 25;10(10):745. doi: 10.3390/genes10100745 (PMC6826356; doi:10.3390/genes10100745)

**Figure S1:** Percentage of reads mapped to transcriptome by Salmon. (A) subchondral bone; (B) articular cartilage. F = fetlock (metatarsophalangeal joint); H = hock (tarsocrural joint); S = stifle (femoropatellar joint).


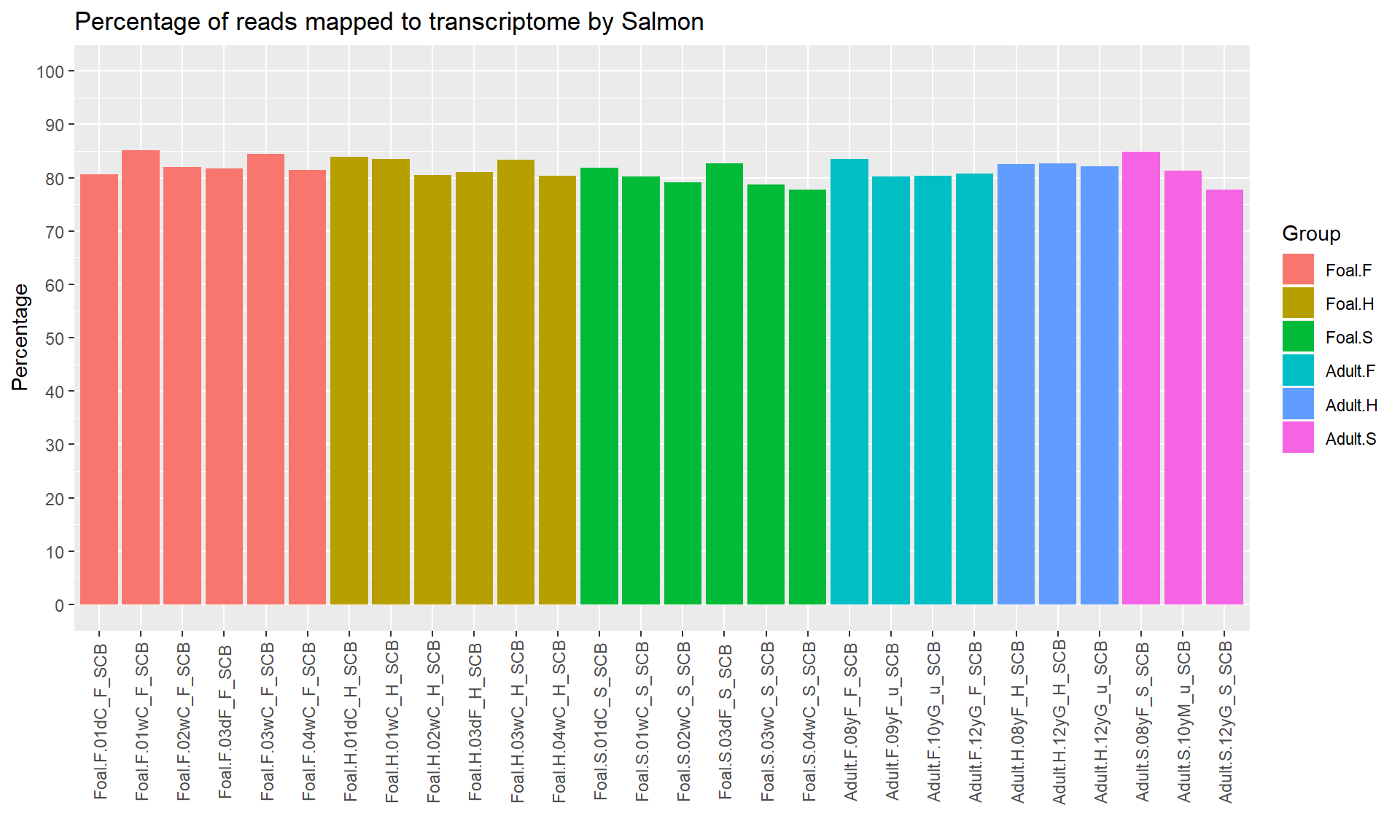

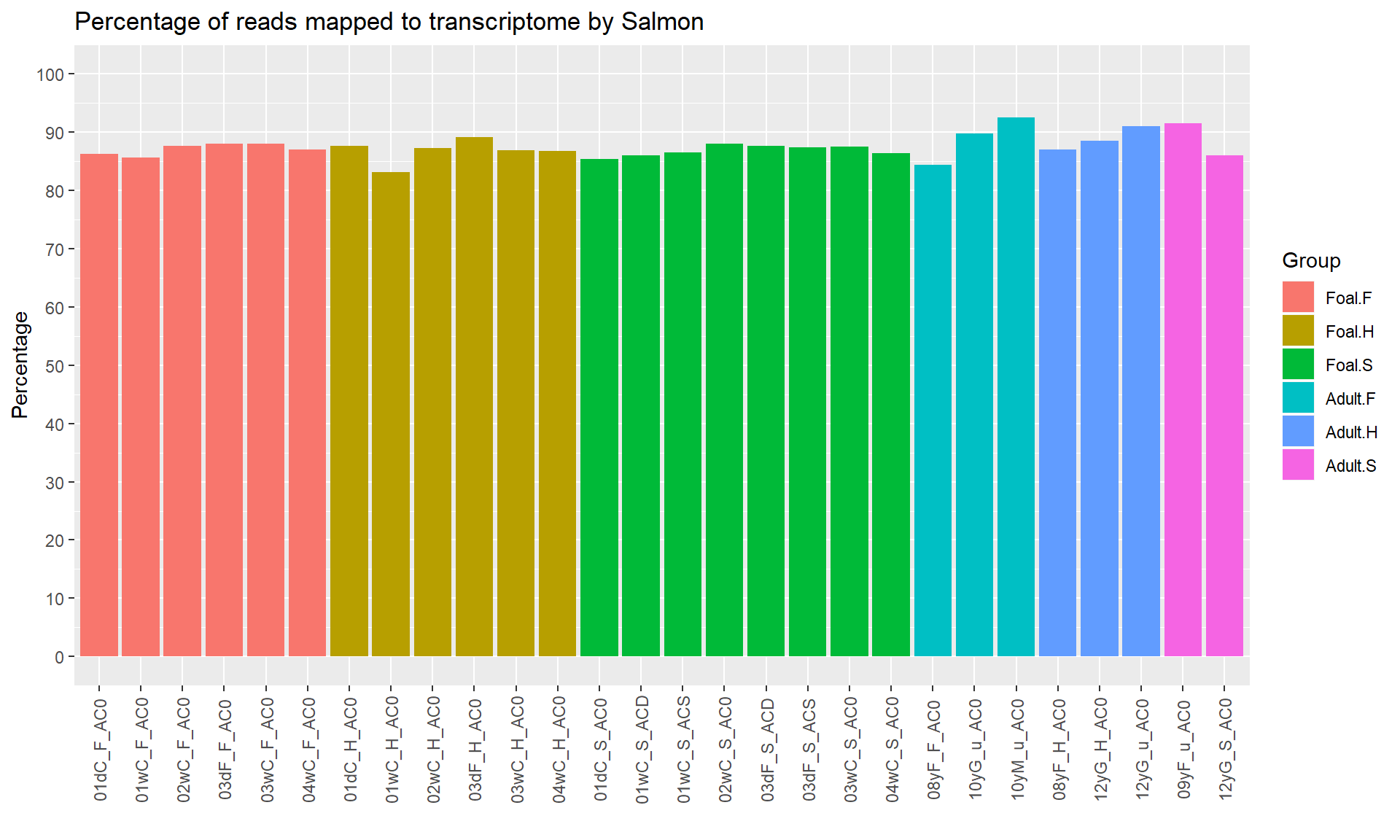


**B**

**A**


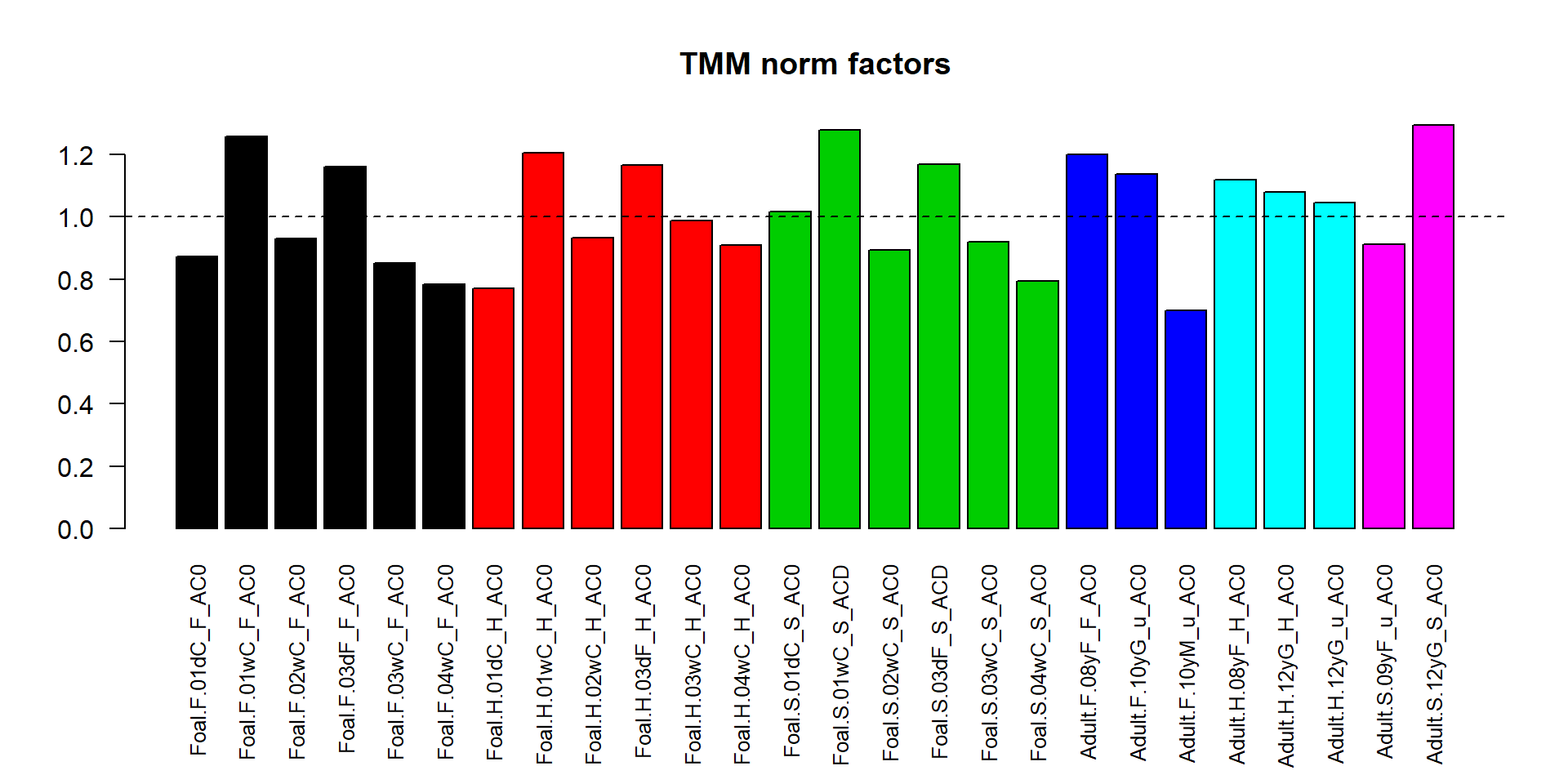
**Figure S2:** TMM normalization factors. (A) subchondral bone; (B) articular cartilage


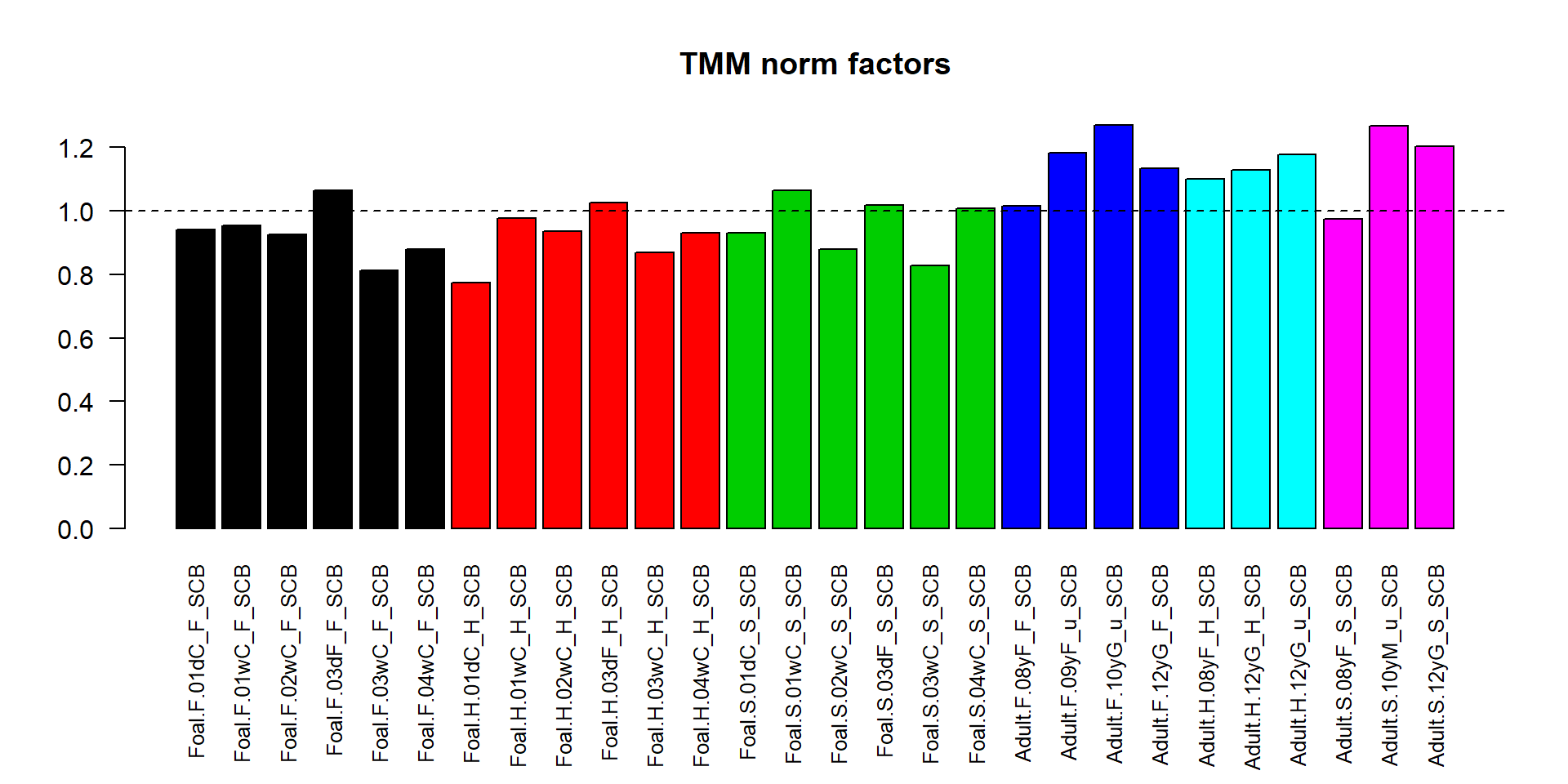


**B**

**A**

**Figure S3:** MDS plot of (A) articular cartilage; and (B) subchondral bone gene expression data prior to the removal of surrogate variables. Compare to **Figure 1** in the main text to appreciate how surrogate variable analysis accounts for nuisance variables.

**
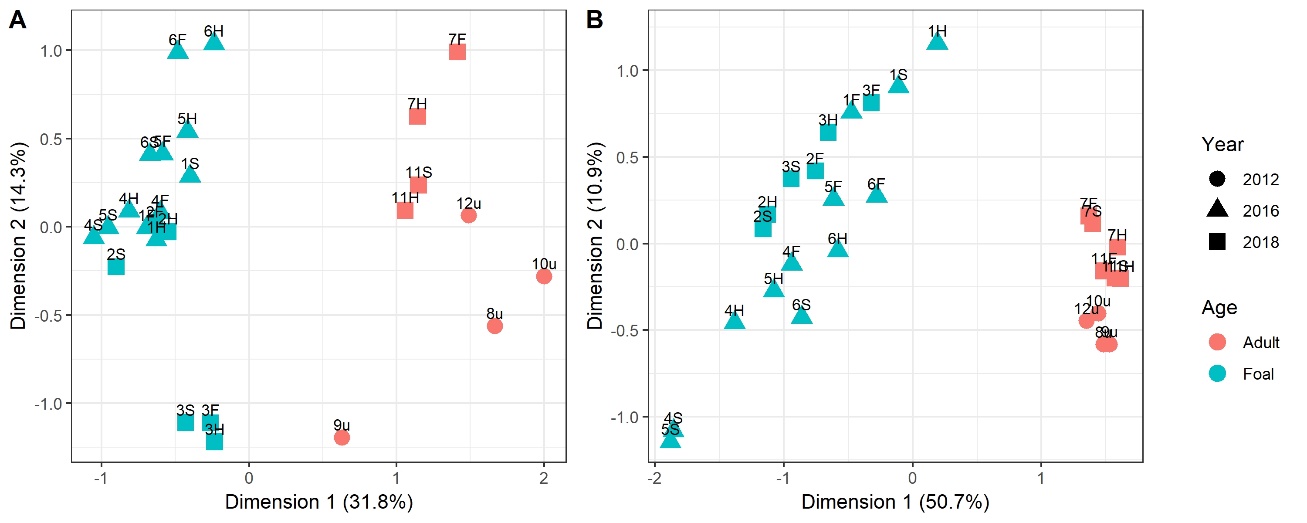
**

**Figure S4:** Boxplots of the estimated surrogate variable values (SV) for AC colored and separated by (A) sequencing year; (B) sex; and (C) individual horse. Because all 4 horses from 2012 each had only one sample, they were combined into the “singles” group in (C).


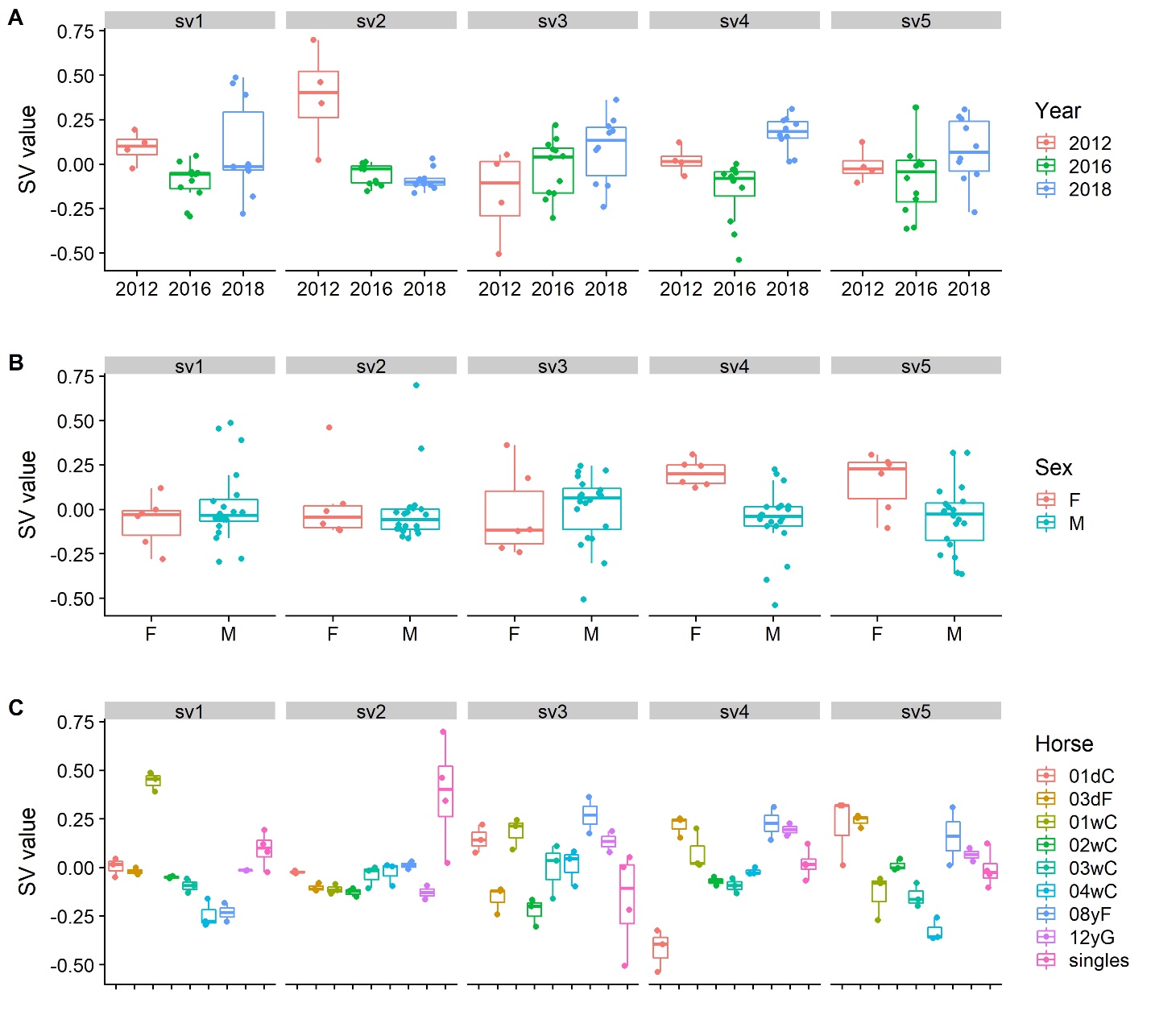


**Figure S5:** Boxplots of the estimated surrogate variable values (SV) for SCB colored and separated by (A) sequencing year; (B) sex; and (C) individual horse. Because all 4 horses from 2012 each had only one sample, they were combined into the “singles” group in (C).


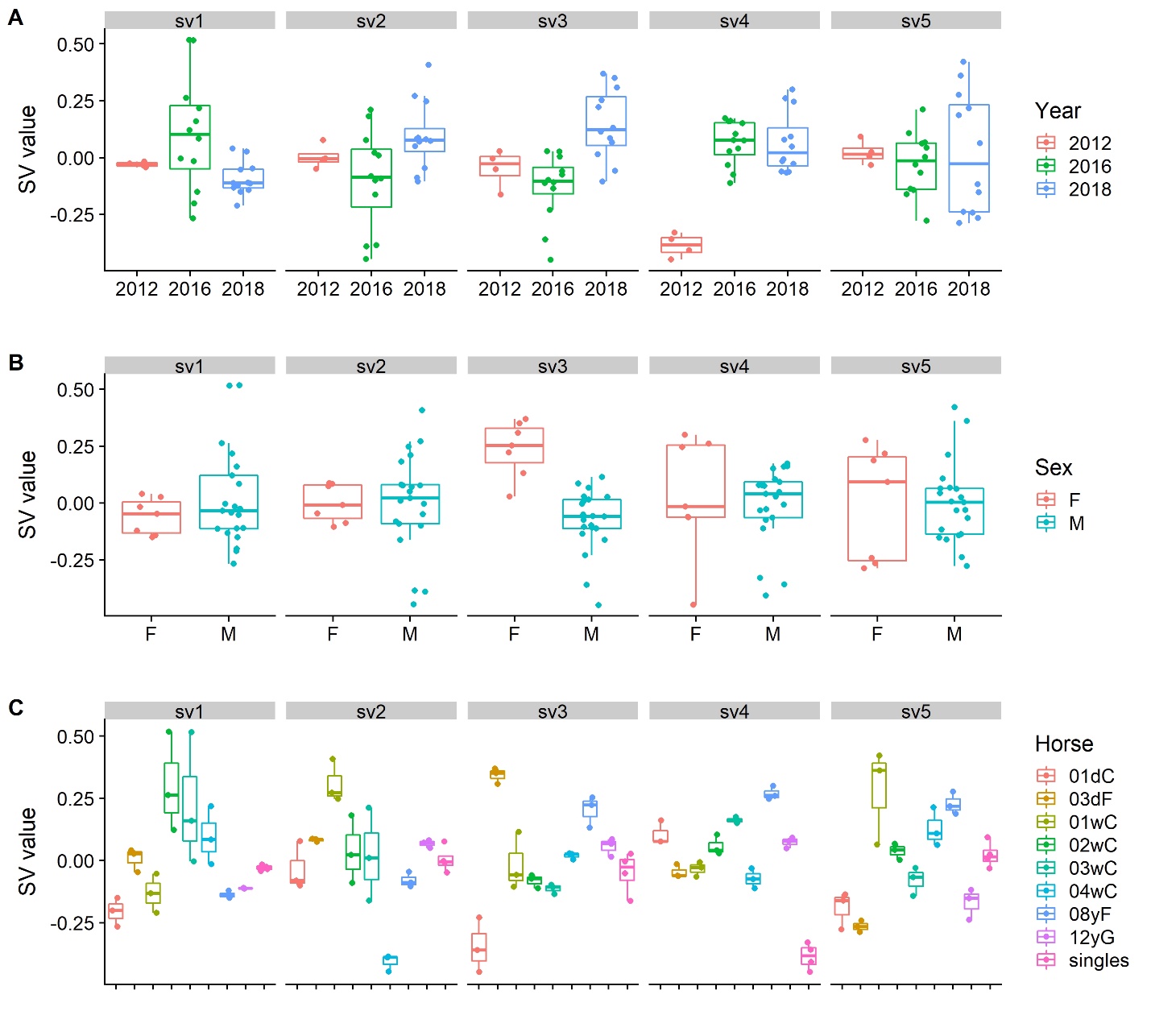

Supplement: Supplementary file 1 [file genes-10-00745-s001.zip › genes-578415-sup/Supplemental Figures.docx]
